# Diet‐Induced Developmental and Morphological Plasticity in a Thelytokous Predatory Mite *Amblyseius herbicolus* (Chant) (Acari: Phytoseiidae)

**DOI:** 10.1002/ece3.72280

**Published:** 2025-10-12

**Authors:** Keshi Zhang, Zhi‐Qiang Zhang

**Affiliations:** ^1^ School of Biological Sciences University of Auckland Auckland New Zealand; ^2^ Manaaki Whenua–Landcare Research Group Bioeconomy Science Institute Auckland New Zealand

**Keywords:** experimental taxonomy, generalist predator, morphological variation, phenotypic plasticity, population differences, prey consumption

## Abstract

Phenotypic plasticity enables organisms to adjust developmental and morphological responses to environmental variations, including prey availability. In this study, we investigated how variation in prey consumption influences immature development and adult morphometric traits in the predatory mite *Amblyseius herbicolus* (Chant). Using two laboratory‐reared populations originating from Auckland and Tauranga, New Zealand, we assessed the relationship between prey consumption and immature development and morphological outcomes. While differences in prey consumption did not significantly alter developmental duration, individuals with higher prey consumption attained a larger size at maturity. Several morphometric traits, including r3 length, primary metapodal plate length, Leg III, and Leg IV, were also significantly influenced by diet, indicating that plasticity extends beyond developmental traits to morphological characteristics in *A. herbicolus*. Population origin also accounted for some variations in developmental and morphometric traits, although it remains unclear whether these differences arose from genetic divergence or prolonged rearing under controlled conditions. This study underscores the importance of actual prey consumption rates—rather than prey availability or density—in shaping morphological variations. It also suggests diet‐induced plasticity may affect phytoseiid mite taxonomy.

## Graphical abstract

This study investigated how variation in prey consumption influences immature development and adult morphometric traits in the predatory mite *Amblyseius herbicolus*. Mites that ate more prey attained a larger size at maturity but did not develop more rapidly. Several morphometric traits were also significantly influenced by diet.

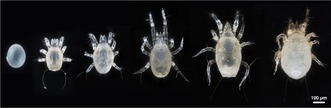

## 1 Introduction

Understanding how organisms respond to environmental variability provides critical insights into evolutionary processes (West‐Eberhard 1989; Enbody et al. 2021). A key mechanism enabling survival and adaptation to such heterogeneity is phenotypic plasticity—the ability of a single genotype to produce multiple phenotypes in response to environmental variation (Blanckenhorn 1998; Pigliucci 2001; Murren et al. 2015). This gene–environment interaction induces intraspecific variation in morphology, physiology, and behaviour, allowing organisms to adjust to fluctuating conditions (Pigliucci 2001; West‐Eberhard 2003).

Among environmental factors, diet plays a crucial role in shaping phenotypic plasticity and the developmental trajectory of individuals, particularly in traits such as age and size at maturity (Ernsting et al. 1992; Han and Dingemanse 2017; Legros et al. 2021). Developmental plasticity, defined as the capacity to modulate growth patterns in response to dietary variation, often results in long‐term and irreversible phenotypic changes (Pigliucci 2001; West‐Eberhard 2003; Taborsky 2017). These life history traits are closely linked to individual fitness and have broad implications for population dynamics (Nilsson‐Örtman and Rowe 2021).

In arthropods, particularly those with determinate growth (i.e., growth stops at maturity), traits such as developmental duration and adult size vary according to diet quantity and quality (Chown and Gaston 2010; Walzer and Schausberger 2011; Plesnar‐Bielak et al. 2013). For instance, individuals tend to develop more rapidly and attain larger sizes under favourable dietary conditions than when dietary conditions are suboptimal (Teder et al. 2014; Nilsson‐Örtman and Rowe 2021). These diet‐induced trade‐offs are widely observed across arthropod taxa and reflect adaptive responses to environmental constraints.

Predatory mites of the family Phytoseiidae (Acari: Mesostigmata) are globally distributed and commonly used as biological control agents in agricultural pest management (Zhang 2003; McMurtry et al. 2015). Species such as *Amblyseius swirskii*, *Neoseiulus cucumeris*, and *Phytoseiulus persimilis* are commercially available and widely employed in biocontrol against pests such as thrips, whiteflies, and spider mites (Amano and Chant 1977; Zhang 2003; McMurtry et al. 2013, 2015). Given their short generation time and the need for sustained population growth in biocontrol systems, the influence of dietary quality and quantity on developmental plasticity in phytoseiids has received considerable attention (Walzer and Schausberger 2011; Han et al. 2022; Yan et al. 2022; Zhang et al. 2024). For example, the specialised spider mite predators *N. californicus* and *P. persimilis* exhibited accelerated development when prey availability was reduced (Walzer and Schausberger 2011). In contrast, the generalist predator *Amblyseius andersoni* showed no such plasticity in developmental time. However, prey availability positively affected the size at maturity of *A. andersoni* , *N. californicus* , and *P. persimilis* (Walzer and Schausberger 2011). These divergent responses possibly reflect their foraging strategies under food limitation: generalists tend to disperse early as immatures to locate alternative food sources, whereas specialists accelerate development to reach maturity early to exploit specific resources.

In phytoseiid mites, morphological traits, such as setal length and pattern, are essential for species identification (Chant and Yoshida‐Shaul 1992; Tixier et al. 2003; Tixier 2012). Functionally, setae—particularly those on the idiosoma—are proposed to aid in navigating prey webbing, facilitating prey capture, avoiding sticky surfaces, interacting with plant trichomes, and enhancing environmental perception and movement (Tixier and Kreiter 2014; Dhooria 2016). In addition to plasticity in age and size at maturity, intraspecific variation in morphological traits, such as the length of specific setae, is common in phytoseiid and other mite species (Ferreira et al. 2021; Afkhami et al. 2024). Environmental factors such as temperature, seasonality, and geographic location have been shown to influence setal length in phytoseiid species such as *Euseius concordis*, *Kampimodromus aberrans*, and *N. californicus* (Tixier et al. 2003, 2008; Lopes et al. 2018). Moreover, variation in diet composition (e.g., pollen vs. prey) affects setal development in *Neoseiulus tunus* and *E. concordis* (Lopes et al. 2018; Ferreira et al. 2021). However, the specific effects of prey availability on morphometric traits in phytoseiid mites remain underexplored.

**FIGURE 1:**
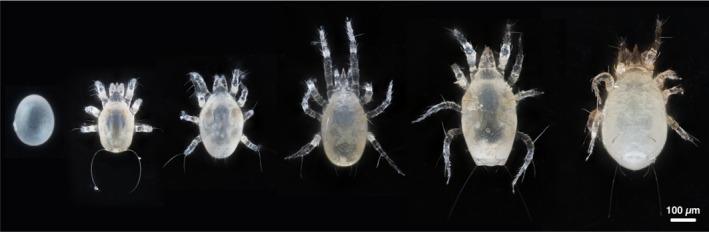
Female *Amblyseius herbicolus* at different life stages/phases. From left to right: Egg, larva, protonymph, deutonymph, newly matured adult, and gravid adult. The larva has six legs, while the nymphs and adults have eight. The photo was taken using a camera adapted to a 20× magnifying lens.

*Amblyseius herbicolus* (Chant) is a generalist phytoseiid predator with a wide distribution and demonstrated potential as a biocontrol agent against various pests, including thrips, psyllids, whiteflies, and mite species (Reis et al. 2007; Cavalcante et al. 2015; Kalile et al. 2021; Lam et al. 2021; Xin and Zhang 2021) (Figure 1). In contrast to most phytoseiid species that reproduce sexually through pseudoarrhenotoky, *A. herbicolus* reproduces asexually through thelytokous parthenogenesis (Hoy 1985; Norton et al. 1993; Zhang and Zhang 2021). It has been suggested by some authors that phenotypic plasticity plays a greater role in asexually reproducing species than in sexually reproducing ones, as it generates phenotypic variation and enhances adaptive potential in response to environmental heterogeneity (Castonguay and Angers 2012; Anastasiadi et al. 2021). Investigating the phenotypic plasticity of *A. herbicolus* could therefore provide important insights into how environmentally induced variation contributes to adaptive capacity in asexual organisms.

This study examined the developmental plasticity of *A. herbicolus* in response to variations in prey availability and assessed whether such plasticity extends to other morphological traits (i.e., morphometric traits relevant to taxonomic identification). We hypothesised that (1) *A. herbicolus* would exhibit a pattern of developmental plasticity similar to *A. andersoni* , reflecting their shared generalist feeding strategies and lifestyle (McMurtry et al. 2013), (2) changes in prey availability would induce variation in morphometric traits, and (3) the effects of prey availability on size at maturity and morphometric traits would be similar between two geographically separated populations. By testing these hypotheses, our findings contribute to a broader understanding of phenotypic plasticity in phytoseiid mites and provide insights into the ecological and taxonomic significance of diet‐induced morphological variation.

## 2 Material and Methods

### 2.1 Culturing

The *A. herbicolus* individuals used in this study originated from two field‐collected populations, sampled in different years and at different locations. One population was established from individuals collected on black nightshade ( *Solanum nigrum* L.) leaves at Manaaki Whenua–Landcare Research, St Johns, Auckland, New Zealand, in early 2023. The second population was derived from individuals collected on avocado ( *Persea americana* Mill.) leaves in an orchard in Te Puna, Tauranga, New Zealand, in early 2024. These two locations are approximately 140 km apart. Over 30 adult females were collected from each site to establish the laboratory colonies. Species identification of *A. herbicolus* was confirmed using the morphological description provided by Ma et al. (2024). Both populations were maintained under laboratory conditions with *ad libitum* access to mixed‐stage dried fruit mites, *Carpoglyphus lactis* (L.) (Acari: Carpoglyphidae), obtained from Bioforce Limited (Karaka, New Zealand). At the time of the experiment, the Auckland population had been maintained in culture for over 1 year, whereas the Tauranga population had been reared for approximately 1 month.

The culturing and experimental rearing set‐ups followed previously published methods, with minor modifications to unit dimensions (Zhang and Zhang 2021; Wang et al. 2024). All cultures and experimental units were kept in acrylic cabinets (100 cm × 55 cm × 100 cm, length × width × height) maintained at 25°C ± 1°C, 80% ± 5% relative humidity, and a 16:8 (light: dark) photoperiod. Cultures were maintained in plastic containers (225 mm × 225 mm × 35 mm) filled with filtered water. A Petri dish (85 mm in diameter) was placed on a black plastic sheet within the container and supported by a piece of water‐soaked sponge (100 mm × 100 mm × 30 mm) to prevent water overflow. The water functioned as both a barrier to prevent mite escape and a hydration source.

The use of frozen prey helps minimize potential interference from prey behaviour and metabolic waste (Xu et al. 2023; Liu et al. 2024a). To standardize diet and eliminate confounding interactions with live prey, this study employed frozen eggs of *C. lactis* , a food source previously validated for use in rearing phytoseiid mites, including *A. herbicolus* (Liu et al. 2024a) and *Phytoseius leaki* (Zhang et al. 2025).

*Carpoglyphus lactis* was cultivated in the Petri dish using a locally sourced mixture consisting of approximately 96% wheat bran (Edmonds, New Zealand), 1% icing sugar (Chelsea, New Zealand), and 3% dry yeast (Edmonds, New Zealand). The bran mixture and *C. lactis* were replenished regularly, and water was refreshed weekly. Frozen *C. lactis* eggs were prepared following the method of Liu et al. (2024b), stored at −18°C for at least 1 week, and thawed at room temperature (c. 25°C) for 30 min before being used in the experiment.

### 2.2 Rearing Cell

**FIGURE 2:**
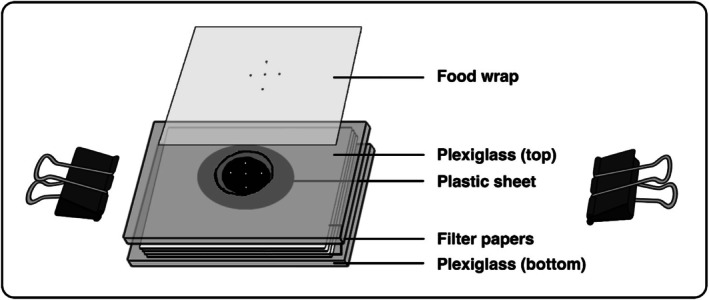
Schematic representation of the experimental cell set‐up used for rearing *Amblyseius herbicolus*.

Rearing cells were used for experimental observations (survival, development, and prey consumption, described below) and consisted of two clear plexiglass slides (37 mm × 24 mm × 2 mm) (Zhang et al. 2024) (Figure 2). The top slide had a centrally located, cone‐shaped hole (10 mm diameter at the top, narrowing to 7 mm at the bottom), forming a chamber with a volume of 115 mm^3^. This chamber was covered with a transparent piece of food wrap to allow visibility, and a black plastic disc (20 mm in diameter) was placed beneath it to provide contrast for viewing mites. Small holes were pierced through both the wrap and disc using a fine insect pin (size 000) to allow air and moisture exchange. A stack of filter papers was placed beneath the disc to provide hydration. The bottom slide supported the moisture reservoir and secured the unit with two foldback metal clips.

### 2.3 Experimental Procedures

1. Approximately 30 nymphs (protonymphs and deutonymphs) of *A. herbicolus* were collected from each laboratory culture (Auckland and Tauranga) to establish cohorts of similar age. The two populations were maintained in separate cultures.
2. Nymphs were provided with *ad libitum* access to mixed‐stage *C. lactis* until oviposition. As *A. herbicolus* reproduces via thelytokous parthenogenesis, mating was not required for oviposition (Zhang and Zhang 2022a).
3. Due to the preference of this species to oviposit on hairy surfaces (Zhang and Zhang 2022b; Liu et al. 2025), sewing threads were placed in the cultures to facilitate egg collection. Approximately 10 days after the establishment (Step 1), eggs of *A. herbicolus* were collected daily and randomly assigned to dietary treatments. Each egg (<16 h old) of *A. herbicolus* was provided with either 20, 30, 40, 50, or 60 thawed *C. lactis* eggs per rearing cell for its entire developmental duration. Each prey availability treatment was replicated 20 times.

### 2.4 Developmental and Morphometric Assessments

This study evaluated both developmental and morphometric responses of *A. herbicolus* eggs to variations in prey availability. Developmental traits included developmental time (egg‐to‐adult duration), prey consumption (total number of eggs consumed during development), and size at maturity. As the dorsal plates of adult phytoseiids, including *A. herbicolus*, are sclerotised and undergo minimal change after reaching maturity, dorsal plate length was used as a measure for size at maturity (Hanna et al. 2023; Ma et al. 2024).

All adults were slide‐mounted for measurements (Walter and Krantz 2009). A total of 66 morphometric traits were measured, including dimensions of the dorsal plate, sternal shield, genital shield, ventrianal shield, setal lengths on the idiosoma, length of the fixed and movable digits, arms of the calyx, length of macrosetae on legs, length of legs, and ratios between specific setae (Table 1) (see Ma et al. 2024 for illustrations). Morphometric traits were measured using a phase‐contrast microscope (Eclipse 90i, Nikon Corporation, Japan) using NIS‐Elements (version 5.10). Magnification levels were adjusted depending on the trait: 200× for dorsal plate and leg measurements, 400× for idiosoma measurements, and 1000× for digit measurements. In some cases, certain traits could not be measured due to suboptimal positioning of specimens on the slide or the loss of morphological features during slide preparation.

**TABLE 1:** Mean ± standard error of the mean (SEM) measurements (all in μm except ratios) of morphometric traits and range (minimum–maximum) of two populations of *Amblyseius herbicolus*. Means followed by asterisks indicate significant differences from *t*‐tests for each character between populations: *p* < 0.05 (*), *p* < 0.01 (**) and *p* < 0.001 (***).

Morphometric traits	Auckland			Tauranga		
	*n*	Mean ± SEM	Range	*n*	Mean ± SEM	Range
Dorsal plate length	63	348.59 ± 1.06***	322.23–368.93	57	341.42 ± 1.26***	319.41–368.56
Dorsal plate width (s4–s4)	63	195.19 ± 0.74	179.18–214.13	56	195.06 ± 1.05	176.88–212.46
j1	63	35.35 ± 0.18	32.10–39.38	57	34.90 ± 0.16	32.32–36.77
j3	63	37.12 ± 0.20	33.49–40.74	57	37.58 ± 0.21	33.25–41.02
j1:j3	63	0.95 ± 0.01**	0.85–1.08	57	0.93 ± 0.00**	0.85–1.03
j4	63	5.71 ± 0.16	3.36–8.17	57	5.71 ± 0.15	3.42–8.57
j5	63	5.69 ± 0.10**	3.99–7.84	57	5.32 ± 0.09**	3.67–7.06
j6	63	7.40 ± 0.13	4.35–9.42	57	7.28 ± 0.10	5.50–8.89
j3:j6	63	5.12 ± 0.10	3.86–8.68	57	5.22 ± 0.08	4.06–6.47
z2	63	12.16 ± 0.15***	8.66–14.74	57	13.17 ± 0.16***	10.61–15.16
z4	63	10.09 ± 0.20*	5.69–14.54	57	9.48 ± 0.18*	5.26–12.54
z5	63	6.38 ± 0.12	4.50–8.66	57	6.17 ± 0.10	4.28–8.21
s4	63	88.82 ± 0.41***	79.39–100.38	57	93.43 ± 0.45***	84.11–100.22
s4:j1	63	2.52 ± 0.02***	2.24–2.83	57	2.68 ± 0.01***	2.48–3.03
r3	63	12.88 ± 0.24	6.36–18.85	57	12.98 ± 0.16	10.10–15.21
J2	63	9.31 ± 0.14**	6.73–11.80	57	8.68 ± 0.12**	6.45–10.80
J5	63	9.18 ± 0.11	6.75–11.37	57	8.97 ± 0.08	7.70–10.22
Z1	63	10.59 ± 0.17	6.30–13.63	56	10.21 ± 0.15	7.29–12.39
Z4	63	92.54 ± 0.45***	85.35–100.37	57	98.64 ± 0.57***	85.61–107.30
Z5	63	238.30 ± 0.75**	217.22–251.74	56	241.70 ± 0.98**	224.74–254.84
Z5:Z4	63	2.58 ± 0.01***	2.37–2.80	56	2.46 ± 0.01***	2.24–2.75
S2	63	10.84 ± 0.16	8.09–13.47	57	11.19 ± 0.16	8.34–13.68
S4	63	10.86 ± 0.15***	7.86–13.63	57	11.64 ± 0.12***	9.79–13.92
S5	63	9.72 ± 0.17	4.66–12.77	57	9.95 ± 0.13	8.23–12.13
R1	63	9.86 ± 0.19*	6.90–15.36	56	9.29 ± 0.17*	6.87–13.74
Sternal shield length	63	77.58 ± 0.34	71.37–86.83	57	78.33 ± 0.35	73.43–86.84
Sternal shield width (st2–st2)	63	71.44 ± 0.19**	68.10–74.94	57	72.50 ± 0.30**	65.92–76.11
st1	63	35.35 ± 0.31*	28.94–40.00	57	34.48 ± 0.22*	30.48–38.64
st2	63	29.49 ± 0.29	22.37–33.94	57	30.01 ± 0.22	26.99–33.48
st3	63	28.34 ± 0.29	22.13–33.18	57	28.44 ± 0.22	23.53–31.93
st4	63	25.87 ± 0.30	20.56–30.69	57	26.60 ± 0.28	21.55–30.92
Epigynal shield length	63	109.00 ± 0.35	101.85–115.78	57	109.35 ± 0.46	102.09–116.91
Epigynal shield width (st5–st5)	63	62.09 ± 0.31***	55.41–68.33	57	58.84 ± 0.35***	46.61–64.51
st5	63	26.91 ± 0.25	21.49–30.31	57	26.87 ± 0.27	20.91–31.03
Ventrianal shield length	63	109.90 ± 0.56	95.05–120.37	57	109.36 ± 0.54	99.76–116.92
Ventrianal shield width (ZV2–ZV2)	63	47.14 ± 0.27	42.95–52.22	57	47.06 ± 0.24	42.65–51.99
JV1	63	20.41 ± 0.19***	17.52–25.20	57	19.09 ± 0.18***	14.32–22.43
ZV2	63	15.90 ± 0.14	13.23–18.87	57	15.46 ± 0.22	12.04–18.74
ZV3	62	8.19 ± 0.17	6.03–11.33	57	8.48 ± 0.16	5.44–10.67
JV2	63	21.42 ± 0.16**	18.01–23.79	57	20.75 ± 0.17**	17.99–23.92
JV2:ZV2	63	1.35 ± 0.01	1.11–1.61	57	1.36 ± 0.02	1.00–1.65
JV4	63	8.44 ± 0.19	5.23–13.99	57	7.64 ± 0.17	4.94–10.22
JV5	63	52.32 ± 0.36	45.30–59.91	57	53.95 ± 0.92	7.74–61.80
ZV1	63	14.03 ± 0.17	11.05–17.91	57	14.07 ± 0.17	11.28–16.57
gv3–gv3	63	25.95 ± 0.20***	21.84–29.52	57	24.74 ± 0.19***	21.54–28.20
Para‐anal seta (Pa)	63	17.74 ± 0.18	14.18–20.68	57	17.63 ± 0.17	14.23–20.47
Post‐anal seta (Pst)	63	16.88 ± 0.19***	12.38–20.20	57	15.69 ± 0.19***	11.21–18.97
Pa:Pst	63	1.06 ± 0.02**	0.79–1.40	57	1.13 ± 0.02**	0.85–1.47
Metapodal plate (primary) length	63	20.23 ± 0.17	16.72–23.85	56	20.35 ± 0.23	15.95–23.88
Metapodal plate (primary) width	63	4.95 ± 0.08*	3.73–7.11	56	4.70 ± 0.08*	3.68–6.54
Spermathecal length	63	28.61 ± 0.28	20.72–33.40	57	28.06 ± 0.26	21.75–32.44
Spermathecal width	63	7.05 ± 0.13**	4.10–9.08	57	6.58 ± 0.11**	4.98–8.53
Fixed digit‐pilus dentilis length	57	10.80 ± 0.12	8.92–12.76	38	10.77 ± 0.12	9.35–12.56
Fixed digit length	63	28.54 ± 0.16***	24.55–32.19	56	29.79 ± 0.14***	28.06–32.45
Movable digit length	63	25.93 ± 0.16***	22.92–28.94	56	26.97 ± 0.16***	24.50–29.31
Leg I	61	460.55 ± 1.66**	415.82–485.41	51	468.12 ± 1.60**	441.44–489.76
I Genu macrosetae	63	45.27 ± 0.39*	33.05–58.90	53	44.27 ± 0.31*	38.64–54.78
Leg II	63	350.55 ± 1.51***	311.84–378.42	57	362.58 ± 1.54***	328.55–390.85
II Genu macrosetae	63	37.12 ± 0.25	34.04–45.25	57	36.73 ± 0.26	33.21–41.34
Leg III	63	349.58 ± 1.63***	327.18–377.59	57	363.24 ± 1.53***	333.08–385.00
III Genu macrosetae	63	44.52 ± 0.30	37.81–55.23	57	43.86 ± 0.34	39.14–50.13
III Tibia macrosetae	63	36.84 ± 0.24	33.39–42.53	57	37.63 ± 0.32	32.02–42.16
Leg IV	63	448.34 ± 2.31***	415.15–512.69	57	467.20 ± 1.90***	427.70–494.46
IV Genu macrosetae	63	115.14 ± 0.58***	99.65–126.58	57	118.62 ± 0.75***	101.86–129.86
IV Tibia macrosetae	63	77.85 ± 0.48***	65.37–89.31	57	82.59 ± 0.51***	71.31–90.31
IV Basitarsus macrosetae	63	66.21 ± 0.40	59.07–73.20	57	66.55 ± 0.68	52.85–75.21

### 2.5 Statistical Analysis

All statistical analyses were conducted in R (R Core Team 2024) using RStudio (version 2023.12.1). Figures were generated using the *ggplot2* package (Wickham 2016). First, survival to adulthood (binary outcome: yes/no) was analysed using logistic regression with prey availability and predator origin as fixed factors, including their interaction. Second, developmental traits (developmental duration and size at maturity) and morphometric traits were analysed using multivariate analyses of covariance (MANCOVAs), followed by univariate ANCOVAs. In these models, prey availability and predator origin were included as fixed factors with their interaction, while prey consumption (i.e., number of prey eggs consumed per predator) was included as a continuous covariate when interactions were not modelled. Linear regressions were then performed separately for each population to assess relationships between prey consumption and developmental duration, size at maturity, and individual morphometric traits. The normality of data on developmental duration and morphometric traits was assessed using the Shapiro–Wilk test, and developmental durations were log‐transformed prior to analysis. Descriptive statistics (means, standard errors of the mean [SEMs], and ranges) were calculated for morphometric traits. Between‐population comparisons for individual traits were conducted using independent‐samples *t*‐tests. Statistical significance was set at *p* < 0.05.

## 3 Results

### 3.1 Survival

**FIGURE 3:**
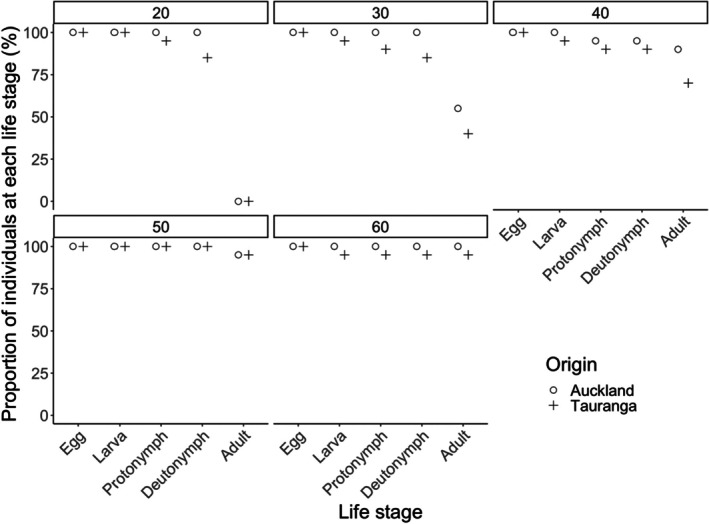
Survival of *Amblyseius herbicolus* (from two different populations) at different life stages when provided with different numbers of frozen *Carpoglyphus lactis* eggs (20, 30, 40, 50, and 60, as indicated by numbers within rectangles).

Immature survival was significantly influenced by prey availability (logistic regression: Wald *χ* ^2^ = 76.904, df = 4, *p* < 0.001), but not by predator origin (Wald *χ* ^2^ = 0.000, df = 1, *p* = 1.000). Specifically, survival rates increased with prey density (Figure 2). No significant interaction was detected between prey availability and predator origin (Wald *χ* ^2^ = 0.578, df = 4, *p* = 0.966). Regardless of prey availability, most individuals reached the deutonymphal stage; however, none of those provided with 20 eggs reached maturity (Figure 3).

### 3.2 Developmental Assessment

Prey availability had no significant overall effect on developmental time or size at maturity (MANCOVA: Pillai trace = 0.081, df = 3, *p* = 0.152). However, both predator origin (Pillai trace = 0.234, df = 1, *p* < 0.001) and prey consumption (Pillai trace = 0.189, df = 1, *p* < 0.001) significantly influenced developmental time or size at maturity. A significant interaction between predator origin and prey consumption was also detected (Pillai trace = 0.166, df = 1, *p* < 0.001).

**FIGURE 4:**
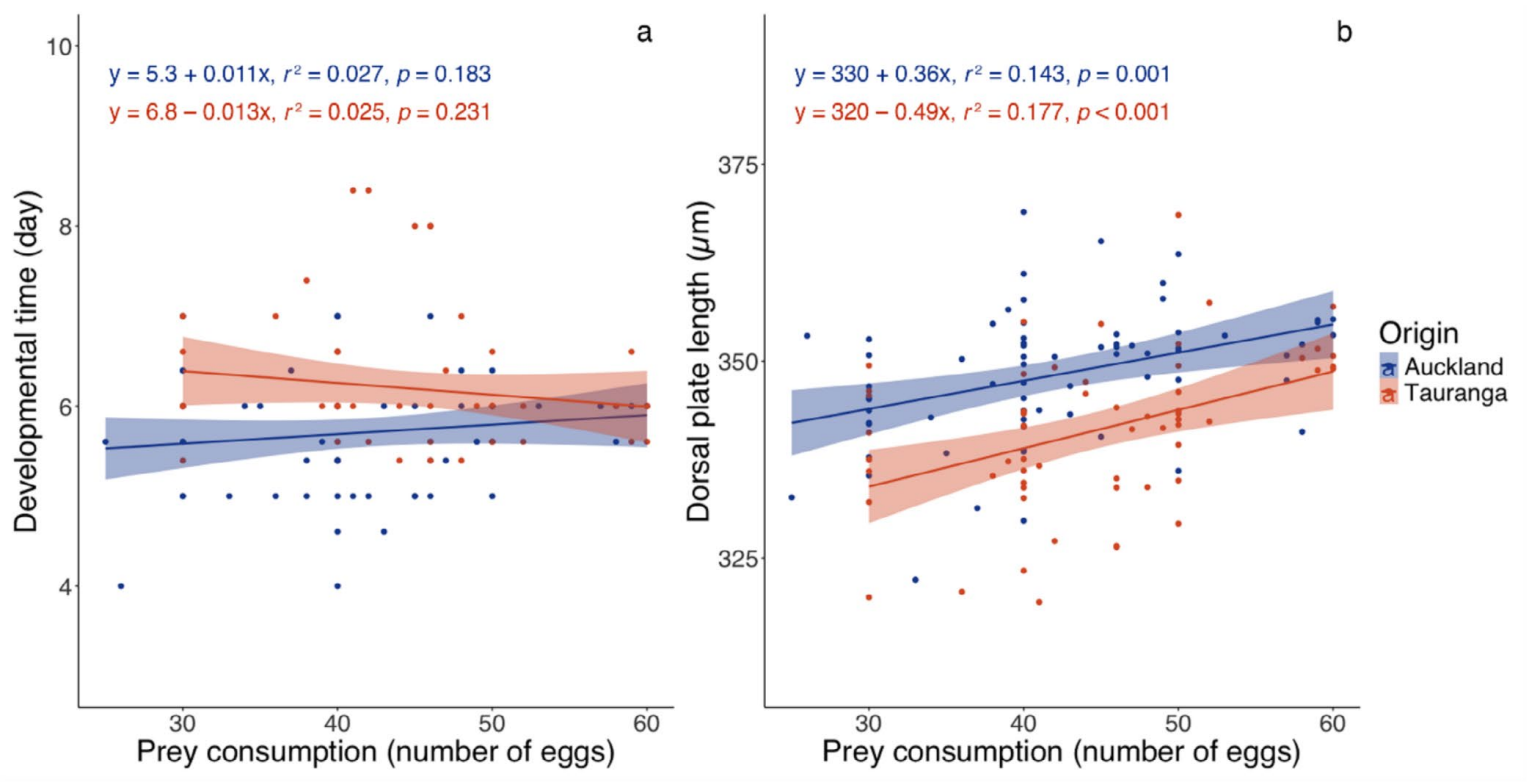
Influence of prey consumption on *Amblyseius herbicolus* development: (a) developmental time; (b) size at maturity (dorsal plate length). The regression line is accompanied by the 95% confidence interval (shaded area). Linear regression equations and test statistics are provided.

Developmental time was not significantly affected by prey availability (ANCOVA: *F*_3,113_ = 0.622, *p* = 0.602) or prey consumption (*F*_1,113_ = 2.161, *p* = 0.144), but was significantly influenced by predator origin (*F*_1,113_ = 19.807, *p* < 0.001). Overall, individuals from Auckland developed faster than those from Tauranga (*t*‐test: *t* = −4.065, df = 126, *p* < 0.001). A significant interaction between prey consumption and predator origin was detected for developmental time (*F*_1,113_ = 21.318, *p* < 0.001), suggesting a population‐specific effect. However, separate linear regressions for each population showed no significant relationship between prey consumption and developmental time (Figure 4a).

In contrast, size at maturity of *A. herbicolus* was significantly influenced by prey availability (ANCOVA: *F*_3,113_ = 2.753, *p* = 0.046), prey consumption (*F*_1,113_ = 17.319, *p* < 0.001), and predator origin (*F*_1,113_ = 25.949, *p* < 0.001). Individuals that consumed more prey developed into larger adults (Figure 3b), and those from Auckland were larger than those from Tauranga (Table 1). A significant interaction between prey consumption and predator origin was also found for size at maturity (*F*_1,113_ = 6.647, *p* = 0.011), with a slightly stronger effect of prey consumption observed in the Tauranga population based on the slope of the regression line (Figure 4b).

### 3.3 Morphometric Assessment

Predator origin had a significant overall effect on morphometric traits (MANCOVA: Pillai trace = 0.980, df = 1, *p* = 0.013), whereas prey availability (Pillai trace = 2.521, df = 3, *p* = 0.897) and prey consumption did not (*Pillai trace* = 0.909, df = 1, *p* = 0.523). Some morphometric traits were significantly longer and/or wider in the Auckland population, while others were greater in the Tauranga population (Table 1); however, 34 traits showed no significant differences between the two populations. ANCOVAs revealed a mixed pattern: some traits were significantly influenced by predator origin, others by prey consumption, and some by neither (Table 2).

**TABLE 2:** Test statistics from analysis of covariance on the influence of prey consumption (Con), prey availability (Ava), and predator origin (Ori) on the morphometric traits of *Amblyseius herbicolus*. Source of variation with significant *p*‐values are given in bold (*p* < 0.05).

Morphometric traits	Source of variation	df	*F*	*p*
Dorsal plate width (s4–s4)	**Con**	1	13.249	**< 0.001**
	Ava	3	0.413	0.744
	Ori	1	0.373	0.543
	Ava × Ori	3	1.048	0.374
j1	Con	1	0.431	0.513
	Ava	3	0.882	0.453
	Ori	1	3.583	0.061
	Ava × Ori	3	0.114	0.952
j3	**Con**	1	4.647	**0.033**
	Ava	3	0.969	0.410
	Ori	1	1.656	0.201
	Ava × Ori	3	0.284	0.837
j1:j3	**Con**	1	5.517	**0.021**
	Ava	3	0.290	0.832
	**Ori**	1	7.026	**0.009**
	Ava × Ori	3	0.504	0.681
j4	Con	1	0.218	0.641
	Ava	3	0.574	0.633
	Ori	1	0.000	0.999
	Ava × Ori	3	2.037	0.113
j5	Con	1	3.707	0.057
	Ava	3	1.284	0.283
	**Ori**	1	8.047	**0.005**
	Ava × Ori	3	1.537	0.209
j6	Con	1	1.977	0.162
	Ava	3	2.501	0.063
	Ori	1	0.477	0.491
	Ava × Ori	3	0.538	0.657
j3:j6	Con	1	1.499	0.223
	Ava	3	2.659	0.052
	Ori	1	0.413	0.522
	Ava × Ori	3	0.768	0.514
z2	Con	1	0.885	0.349
	Ava	3	1.109	0.349
	**Ori**	1	22.712	**< 0.001**
	**Ava × Ori**	3	3.510	**0.018**
z4	Con	1	2.544	0.114
	Ava	3	0.408	0.747
	**Ori**	1	4.246	**0.042**
	Ava × Ori	3	0.217	0.884
z5	Con	1	1.146	0.287
	Ava	3	0.543	0.654
	Ori	1	1.769	0.186
	Ava × Ori	3	0.608	0.611
s4	**Con**	1	7.640	**0.007**
	Ava	3	0.554	0.647
	**Ori**	1	56.629	**< 0.001**
	Ava × Ori	3	1.623	0.188
s4:j1	**Con**	1	7.616	**0.007**
	Ava	3	1.945	0.127
	**Ori**	1	55.995	**< 0.001**
	Ava × Ori	3	0.653	0.583
r3	**Con**	1	10.611	**0.001**
	Ava	3	1.159	0.329
	Ori	1	0.004	0.951
	Ava × Ori	3	2.266	0.085
J2	Con	1	0.059	0.808
	Ava	3	1.061	0.369
	**Ori**	1	9.436	**0.003**
	Ava × Ori	3	0.032	0.992
J5	Con	1	1.036	0.311
	Ava	3	0.144	0.933
	Ori	1	2.852	0.094
	Ava × Ori	3	1.367	0.257
Z1	Con	1	0.047	0.829
	Ava	3	1.059	0.369
	Ori	1	2.763	0.099
	Ava × Ori	3	1.677	0.176
Z4	Con	1	0.103	0.748
	Ava	3	0.754	0.522
	**Ori**	1	74.229	**< 0.001**
	Ava × Ori	3	0.550	0.649
Z5	Con	1	0.670	0.415
	Ava	3	0.921	0.433
	**Ori**	1	8.020	**0.006**
	Ava × Ori	3	2.018	0.116
Z5:Z4	Con	1	0.003	0.956
	Ava	3	0.230	0.875
	**Ori**	1	43.345	**< 0.001**
	Ava × Ori	3	0.970	0.410
S2	**Con**	1	5.120	**0.026**
	Ava	3	0.754	0.523
	Ori	1	1.714	0.193
	Ava × Ori	3	0.734	0.534
S4	Con	1	1.945	0.166
	Ava	3	0.475	0.701
	**Ori**	1	14.877	**< 0.001**
	Ava × Ori	3	1.341	0.265
S5	**Con**	1	4.159	**0.044**
	Ava	3	2.518	0.062
	Ori	1	0.527	0.469
	Ava × Ori	3	1.029	0.383
R1	Con	1	1.367	0.245
	Ava	3	1.515	0.215
	**Ori**	1	5.192	**0.025**
	Ava × Ori	3	1.433	0.237
Sternal shield length	Con	1	2.680	0.104
	Ava	3	1.286	0.283
	Ori	1	2.025	0.158
	Ava × Ori	3	0.180	0.910
Sternal shield width (st2–st2)	**Con**	1	14.262	**< 0.001**
	Ava	3	0.428	0.733
	**Ori**	1	6.774	**0.011**
	Ava × Ori	3	0.512	0.675
st1	Con	1	0.095	0.758
	Ava	3	1.574	0.200
	**Ori**	1	5.395	**0.022**
	Ava × Ori	3	0.861	0.464
st2	Con	1	2.114	0.149
	Ava	3	1.980	0.121
	Ori	1	1.863	0.175
	Ava × Ori	3	0.550	0.649
st3	Con	1	0.689	0.408
	Ava	3	0.067	0.977
	Ori	1	0.030	0.863
	Ava × Ori	3	0.834	0.478
st4	**Con**	1	8.220	**0.005**
	**Ava**	3	3.083	**0.030**
	Ori	1	3.158	0.078
	Ava × Ori	3	0.679	0.567
Epigynal shield length	**Con**	1	16.991	**< 0.001**
	**Ava**	3	2.985	**0.034**
	Ori	1	0.130	0.719
	Ava × Ori	3	2.177	0.095
Epigynal shield width (st5–st5)	Con	1	0.344	0.559
	Ava	3	0.973	0.408
	**Ori**	1	50.789	**< 0.001**
	Ava × Ori	3	1.410	0.244
st5	Con	1	0.164	0.686
	**Ava**	3	3.127	**0.029**
	Ori	1	0.009	0.927
	Ava × Ori	3	0.039	0.990
Ventrianal shield length	**Con**	1	10.205	**0.002**
	Ava	3	1.128	0.341
	Ori	1	0.897	0.346
	Ava × Ori	3	0.497	0.685
Ventrianal shield width (ZV2–ZV2)	**Con**	1	6.560	**0.012**
	Ava	3	0.497	0.685
	Ori	1	0.318	0.574
	Ava × Ori	3	0.354	0.786
JV1	Con	1	0.001	0.973
	Ava	3	2.159	0.097
	**Ori**	1	24.002	**< 0.001**
	Ava × Ori	3	0.008	0.999
ZV2	Con	1	0.382	0.538
	Ava	3	0.747	0.526
	Ori	1	2.892	0.092
	Ava × Ori	3	1.922	0.130
ZV3	**Con**	1	5.230	**0.024**
	Ava	3	0.682	0.565
	Ori	1	1.183	0.279
	Ava × Ori	3	1.014	0.390
JV2	**Con**	1	7.029	**0.009**
	Ava	3	2.273	0.084
	**Ori**	1	11.427	**0.001**
	Ava × Ori	3	0.808	0.492
JV2:ZV2	Con	1	0.813	0.369
	Ava	3	1.925	0.130
	Ori	1	0.022	0.883
	**Ava × Ori**	3	2.878	**0.039**
JV4	Con	1	0.158	0.692
	Ava	3	0.133	0.940
	**Ori**	1	9.980	**0.002**
	Ava × Ori	3	0.668	0.573
JV5	Con	1	0.113	0.737
	Ava	3	0.307	0.820
	Ori	1	2.899	0.091
	Ava × Ori	3	0.557	0.644
ZV1	Con	1	0.377	0.540
	Ava	3	0.744	0.528
	Ori	1	0.094	0.760
	Ava × Ori	3	0.731	0.536
gv3–gv3	Con	1	3.299	0.072
	Ava	3	0.557	0.644
	**Ori**	1	20.580	**< 0.001**
	Ava × Ori	3	0.121	0.948
Para‐anal seta (Pa)	**Con**	1	7.672	**0.007**
	Ava	3	1.475	0.225
	Ori	1	0.568	0.453
	Ava × Ori	3	0.867	0.460
Post‐anal seta (Pst)	Con	1	0.263	0.609
	Ava	3	0.549	0.650
	**Ori**	1	21.428	**< 0.001**
	Ava × Ori	3	0.713	0.546
Pa:Pst	Con	1	1.818	0.180
	Ava	3	1.069	0.366
	**Ori**	1	10.553	**0.002**
	Ava × Ori	3	1.062	0.368
Metapodal plate (primary) length	**Con**	1	9.728	**0.002**
	Ava	3	0.143	0.934
	Ori	1	0.000	0.997
	Ava × Ori	3	0.398	0.755
Metapodal plate (primary) width	**Con**	1	6.493	**0.012**
	Ava	3	0.181	0.909
	Ori	1	3.421	0.067
	Ava × Ori	3	0.506	0.679
Spermathecal width	Con	1	0.049	0.825
	Ava	3	0.421	0.739
	Ori	1	1.696	0.196
	Ava × Ori	3	0.770	0.513
Spermathecal length	Con	1	0.017	0.896
	Ava	3	1.721	0.167
	**Ori**	1	6.590	**0.012**
	Ava × Ori	3	0.119	0.949
Fixed digit‐pilus dentilis length	Con	1	0.009	0.923
	Ava	3	1.205	0.313
	Ori	1	0.153	0.697
	Ava × Ori	3	1.122	0.345
Fixed digit length	Con	1	3.791	0.054
	Ava	3	0.620	0.604
	**Ori**	1	31.255	**< 0.001**
	Ava × Ori	3	1.360	0.259
Movable digit length	Con	1	3.201	0.076
	Ava	3	0.889	0.449
	**Ori**	1	17.597	**< 0.001**
	Ava × Ori	3	0.295	0.829
Leg I	**Con**	1	10.023	**0.002**
	Ava	3	0.132	0.941
	**Ori**	1	8.033	**0.006**
	Ava × Ori	3	0.543	0.654
I Genu macrosetae	Con	1	0.004	0.947
	Ava	3	1.790	0.153
	Ori	1	3.798	0.054
	Ava × Ori	3	1.807	0.150
Leg II	**Con**	1	12.139	**0.001**
	Ava	3	0.095	0.963
	**Ori**	1	27.087	**< 0.001**
	Ava × Ori	3	0.355	0.786
II Genu macrosetae	Con	1	2.259	0.136
	Ava	3	0.955	0.417
	Ori	1	1.522	0.220
	Ava × Ori	3	0.750	0.525
Leg III	**Con**	1	13.468	**< 0.001**
	Ava	3	1.014	0.390
	**Ori**	1	32.663	**< 0.001**
	Ava × Ori	3	0.502	0.681
III Genu macrosetae	Con	1	1.761	0.187
	Ava	3	1.641	0.184
	Ori	1	2.270	0.135
	Ava × Ori	3	0.656	0.581
III Tibia macrosetae	Con	1	1.372	0.244
	Ava	3	1.638	0.185
	**Ori**	1	4.236	**0.042**
	Ava × Ori	3	2.452	0.067
Leg IV	**Con**	1	15.923	**< 0.001**
	Ava	3	0.562	0.641
	**Ori**	1	35.380	**< 0.001**
	Ava × Ori	3	0.581	0.629
IV Genu macrosetae	Con	1	1.986	0.162
	Ava	3	0.161	0.923
	**Ori**	1	12.566	**0.001**
	Ava × Ori	3	0.167	0.919
IV Tibia macrosetae	Con	1	1.323	0.253
	Ava	3	0.763	0.517
	**Ori**	1	42.850	**< 0.001**
	Ava × Ori	3	0.087	0.967
IV Basitarsus macrosetae	Con	1	0.448	0.505
	Ava	3	0.618	0.605
	Ori	1	0.279	0.598
	Ava × Ori	3	0.979	0.405

A significant interaction between predator origin and prey consumption was detected for overall morphometric variation (MANCOVA: *F*_66,19_ = 2.561, *p* = 0.014), with specific traits also showing significant interactions in ANCOVAs (Table 2). Linear regression analyses showed that 15 traits in the Tauranga population (dorsal plate width, j3 length, j1:j3 ratio, z2 length, z5 length, sternal shield width, st4 length, epigynal shield width, ventrianal shield length, ventrianal shield width, ZV3 length, fixed digit‐pilus dentilis length, and lengths of Leg I, Leg II, and Leg II genu macrosetae) were significantly affected by prey consumption (Table 3). No such effects were observed for these traits in the Auckland population. Conversely, four traits (S2 length, JV2 length, JV2:ZV2 ratio, and para‐anal seta length [Pa length]) were significantly influenced by prey consumption in the Auckland population, but not in the Tauranga population (Table 3). Only five traits (dorsal plate length, r3 length, primary metapodal plate length, Leg III, and Leg IV) were significantly influenced by prey consumption in both populations (Figure 4b, Table 3). All traits exhibited positive correlations with prey consumption, except the j1:j3 ratio and fixed digit‐pilus dentilis length, which were negatively correlated.

**TABLE 3:** Test statistics from linear regressions on the influence of prey consumption on morphometric traits of *Amblyseius herbicolus*. Traits are grouped based on whether the effect was significant in both populations or in one population only (*p* < 0.05).

Population(s) with significant effect	Morphometric traits	Origin	*F*	*p*	df
Both populations	r3	Auckland	5.898	0.018	1, 61
		Tauranga	4.312	0.043	1, 55
	Metapodal plate (primary) length	Auckland	4.087	0.048	1, 61
		Tauranga	5.985	0.018	1, 54
	Leg III	Auckland	4.189	0.045	1, 61
		Tauranga	4.509	0.038	1, 55
	Leg IV	Auckland	4.144	0.046	1, 61
		Tauranga	7.393	0.009	1, 55
Auckland only	S2	Auckland	6.409	0.014	1, 61
	JV2	Auckland	8.304	0.005	1, 61
	JV2:ZV2	Auckland	4.362	0.041	1, 61
	Para‐anal seta	Auckland	5.718	0.020	1, 61
Tauranga only	Dorsal plate width	Tauranga	14.085	< 0.001	1, 54
	j3	Tauranga	4.677	0.035	1, 55
	j1:j3	Tauranga	6.243	0.015	1, 55
	z2	Tauranga	6.688	0.012	1, 55
	z5	Tauranga	5.156	0.027	1, 55
	Sternal shield width (st2–st2)	Tauranga	8.9	0.004	1, 55
	st4	Tauranga	4.063	0.049	1, 55
	Epigynal shield width (st5–st5)	Tauranga	27.469	< 0.001	1, 55
	Ventrianal shield length	Tauranga	14.75	< 0.001	1, 55
	Ventrianal shield width (ZV2–ZV2)	Tauranga	16.377	< 0.001	1, 55
	ZV3	Tauranga	6.386	0.014	1, 55
	Fixed digit‐pilus dentilis length	Tauranga	4.944	0.033	1, 36
	Leg I	Tauranga	4.666	0.036	1, 49
	Leg II	Tauranga	9.261	0.004	1, 55
	Leg II Genu macrosetae	Tauranga	6.039	0.017	1, 55

## 4 Discussion

Phenotypic plasticity enables organisms to adjust developmental and morphological traits in response to environmental variability. In this study, we examined how prey consumption influences developmental duration and morphometric traits in two laboratory populations of *A. herbicolus* reared under controlled conditions. We hypothesised that (1) higher prey consumption during development would result in larger adult size at maturity, (2) this effect would extend to morphometric traits, and (3) the responses would be consistent between populations. The first hypothesis was supported: *A. herbicolus* exhibited diet‐induced phenotypic plasticity similar to that previously reported for *A. andersoni* , whereby increased prey consumption resulted in larger body size without affecting developmental time (Walzer and Schausberger 2011). The second hypothesis was partially supported, as prey consumption influenced some morphometric traits, although the effect was not consistent across all traits measured. Finally, the third hypothesis was not supported, as clear population‐level differences were observed in diet‐induced developmental and morphological plasticity.

As mentioned above, variation in prey availability and consumption did not significantly influence the developmental time of *A. herbicolus*, which aligns with previous studies on generalist predators such as *A. andersoni* (Walzer and Schausberger 2011). For individuals provided with fewer prey items, all available prey eggs were consumed early in development (K. Zhang, pers. obs.), which may account for the absence of a detectable effect on developmental time. Future studies could examine whether extending the feeding interval can reveal differences in developmental duration. Moreover, conducting observations at shorter time intervals may yield more precise estimates of developmental duration, potentially detecting subtle diet‐induced effects not captured with the current monitoring resolution.

Although prey availability and consumption did not influence developmental time in *A. herbicolus*, they significantly affected size at maturity, with increased prey intake resulting in a larger adult body size. This result is consistent with previous studies on other phytoseiid predators and insect species (Blanckenhorn 2009; Walzer and Schausberger 2011). However, significant population‐level differences in developmental traits were observed. Individuals from Auckland exhibited a shorter developmental duration and attained a larger size at maturity when fed an equivalent diet. Since size at maturity is influenced by both developmental duration and growth rate (Abrams et al. 1996), these findings suggest that *A. herbicolus* from Auckland may exhibit a higher growth rate under the experimental conditions compared to those from Tauranga.

The influence of diet extended beyond overall size to specific morphometric traits, suggesting broader phenotypic plasticity. This aligns with the notion that physiological trade‐offs during development can lead to differential investment in morphological traits (Stearns 1989; Garland 2014). However, the ecological or fitness consequences of these morphological variations in *A. herbicolus* require further investigation. In Phytoseiidae, where individuals lack functional eyes, intraspecific interactions rely heavily on chemical and tactile cues (Kawasaki et al. 2009; Hoy et al. 2016; Zhang and Zhang 2022a, 2022b). Previous studies have shown that larger *P. persimilis* individuals of both sexes have greater mating success than their smaller counterparts (Walzer and Schausberger 2013, 2015). These findings raise the question of whether specific morphological traits in the Phytoseiidae might reflect individual nutritional status and influence mate recognition, particularly in species where body size affects reproductive success.

Despite the observed population‐level differences in developmental and morphological plasticity, two potential confounding factors must be considered: population origin and duration of laboratory rearing. The Auckland population had been maintained in laboratory conditions for over a year, while the Tauranga population had only been reared for approximately 1 month. The observed differences may thus stem from genetic divergence, environmental influences, or both. Local adaptation, founder effects, genetic drift, or transgenerational epigenetic inheritance may all contribute to population‐specific responses (Sgrò and Partridge 2000; Kolbe et al. 2012; Santos et al. 2012; Pfennig and Servedio 2013; Sparks et al. 2024). For instance, geographically distinct populations of the spider *Nephila clavipes* exhibit distinct growth rates (Higgins 1992), and similar interpopulation variation in morphometric traits has been reported among phytoseiid mites (Tixier et al. 2003, 2008). Additionally, long‐term laboratory rearing under uniform conditions may impose selective pressures that alter life history traits and reduce phenotypic variability (Sgrò and Partridge 2000; Li et al. 2014; LaCava et al. 2023). Therefore, the Auckland population's longer laboratory acclimation may have influenced its developmental responses and reduced morphological plasticity relative to the recently established Tauranga population. Further research is needed to determine whether these differences arise from intrinsic genetic divergence or laboratory‐induced selection.

Although several morphometric traits were statistically different between populations (*p* < 0.05), there were substantial overlaps in trait ranges (minimum–maximum), and it remains unclear whether these differences have taxonomic relevance. Notably, as individuals may not consume all prey provided, prey consumption constitutes a more accurate measure than prey availability when assessing developmental plasticity in *A. herbicolus*. Our results highlight the importance of quantifying actual prey consumption in studies evaluating developmental and morphological plasticity (Han et al. 2022).

In summary, variation in prey consumption significantly affected size at maturity and several morphological traits in *A. herbicolus*. The variation in these morphological traits may reflect their ecological and functional importance that should be examined further. Future work should investigate how body size influences predation efficiency, reproductive output, and stress tolerance, as well as how variation in leg length affects prey capture and locomotion. While predator origin also contributed to morphometric variation, further studies would be required to disentangle the relative contributions of population differentiation and laboratory acclimatisation. An enhanced understanding of how diet shapes morphological plasticity in *A. herbicolus* may offer broader insights into its ecological adaptability and potential taxonomic implications.

## Author Contributions

**Keshi Zhang:** conceptualization (lead), data curation (lead), formal analysis (lead), investigation (lead), methodology (lead), validation (lead), visualization (lead), writing – original draft (lead), writing – review and editing (lead). **Zhi‐Qiang Zhang:** conceptualization (supporting), funding acquisition (lead), project administration (lead), supervision (lead), writing – original draft (supporting), writing – review and editing (supporting).

## Conflicts of Interest

The authors declare no conflicts of interest.

## Supporting information


**Appendix S1:** ece372280‐sup‐0001‐AppendixS1.csv.

**Appendix S2:** ece372280‐sup‐0002‐AppendixS2.csv.

**Appendix S3:** ece372280‐sup‐0003‐AppendixS3.csv.

## Data Availability

Data and codes used are all submitted.
